# Stereotactic Body Radiation Therapy in the Management of Upper GI Malignancies

**DOI:** 10.3390/biomedicines6010007

**Published:** 2018-01-03

**Authors:** Leila Tchelebi, Nicholas Zaorsky, Heath Mackley

**Affiliations:** Penn State Health Milton S. Hershey Medical Center, Hershey, PA 17033, USA; nzaorsky@pennstatehealth.psu.edu (N.Z.); hmackley@pennstatehealth.psu.edu (H.M.)

**Keywords:** stereotactic body radiation therapy, immunotherapy, biomarkers

## Abstract

The role of external beam radiation therapy (EBRT) in the management of upper gastrointestinal malignancies is constantly evolving. As radiation therapy techniques improve and are able to deliver more ablative doses of radiotherapy while sparing healthy tissue, radiation can be applied to a wider range of clinical scenarios. Stereotactic body radiation therapy (SBRT) allows a high dose of radiation to be delivered to a highly conformal treatment volume in a short amount of time. Another potential advantage of SBRT is its ability to increase tumor immunogenicity, while also having less of an immunosuppressive effect on the patient, as compared to conventionally fractionated radiation therapy. In so doing, SBRT may potentiate the effects of immune therapy when the two treatments are combined, thus improving therapeutic outcomes. This article provides an overview of the role of SBRT in the management of upper gastrointestinal GI malignancies and the emerging data on immune biomarkers and SBRT, with a focus on pancreatic and liver cancer.

## 1. Introduction

The role of external beam radiation therapy in the management of upper gastrointestinal (GI) malignancies is constantly evolving. Surgery has historically been the cornerstone of treatment for a majority of these cancers, particularly for pancreatic and hepatobiliary malignancies, with radiation reserved for palliation of symptoms. However, as radiation therapy techniques improve and are able to deliver more ablative doses of radiotherapy while sparing healthy tissue, radiation can be applied to a wider range of clinical scenarios.

Stereotactic body radiation therapy (SBRT) is a technique that allows a high dose of radiation to be delivered to a highly conformal treatment volume in a short amount of time. This results in a number of advantages. First, it allows for treatment of a higher biologically effective dose (BED), thus improving local tumor control. Second, it allows for a shorter overall treatment time which is both more convenient for patients and treating facilities and, more importantly, prevents delays in systemic treatment and/or surgery. Finally, given the highly conformal nature of SBRT, it allows for increased sparing of adjacent organs at risk (OARs) [1]. While the role of SBRT in the treatment of organs organized in series, such as the esophagus, is not well defined, its use in treating those organized in parallel, such as the liver and pancreas, has been established. This article therefore seeks to review the role of SBRT in the management of upper GI malignancies with a focus on pancreatic and liver cancer.

Another potential advantage of SBRT is its ability to increase tumor immunogenicity while having less of an immunosuppressive effect on the patient, as compared to conventionally fractionated radiation therapy [2]. In so doing, SBRT may potentiate the effects of immune therapy when the two treatments are combined, thus improving therapeutic outcomes. While the available data on tumor biomarkers and SBRT is in its infancy, it is hypothesis-generating and will also be reviewed in this article.

## 2. SBRT for Pancreatic Cancer

Pancreatic cancer is the 4th leading cause of cancer death in the United States [2]. Currently, surgery is considered the only curative treatment; however, less than 20% of patients are operable at the time of diagnosis [3,4]. Radiation therapy, therefore, plays a role in both the preoperative and definitive management of pancreatic malignancies. However, because the pancreas is considered to be radio-resistant, higher radiation doses are required for tumor control [5]. Indeed, studies have shown that delivering a BED of greater than 70 Gy is associated with improved survival for pancreatic cancer [6]. This poses a challenge for standard radiotherapy treatment planning due to the close proximity of highly radiosensitive organs such as the liver, duodenum, and stomach. SBRT in the management of pancreatic cancer is therefore a highly appealing treatment modality which has been well studied [7,8,9,10,11,12,13,14,15,16,17,18,19,20,21,22,23,24,25,26,27].

### 2.1. The Neoadjuvant Setting

The close proximity of the pancreas to critical vascular structures makes it technically challenging to achieve microscopically negative (R0) resections. The presence of positive margins following resection is associated with inferior outcomes [27,28,29,30,31]. Radiation therapy has, therefore, been used in the preoperative setting to downsize tumors intimately associated with vascular structures so as to increase the rate of negative margins at the time of surgery. Given the advantages of SBRT outlined above, there is growing interest in utilizing this technique in the neoadjuvant setting.

In 2015, the Moffitt Center published its experience with SBRT for borderline resectable pancreatic cancer [32]. This was a retrospective institutional review of all patients treated neoadjuvantly with SBRT at their institution. It included 159 patients, the majority of which (110) had borderline resectable disease (BRPC), with the remaining patients (49) having locally advanced unresectable pancreatic cancer (LAPC). Patients were treated with multi-agent systemic chemotherapy, followed by SBRT to a median dose of 40 Gy in five fractions and then surgery in patients who were deemed resectable after neoadjuvant therapy.

Ultimately, 51% of BRPC and 10% of LAPC patients were able to undergo surgery. The R0 resection rate in these patients was 96% and 100%, respectively. Seven percent of patients had a pathologic complete response and none of these patients relapsed at a median follow-up of 14 months. The overall survival and progression free survival rates were 19 months and 12 months, respectively, for those with BRPC, and 15 months and 13 months, respectively, for those with LAPC. Remarkably, the overall survival rate for patients who received neoadjuvant treatment and were then able to undergo surgery was 34 months, versus only 14 months for those who received chemotherapy and radiation but remained unresectable. Overall survival rates for up-front resectable pancreatic cancer patients, historically thought to have the best survival outcomes, has not exceeded 30 months in national randomized controlled trials, including the recently published ESPAC-4 trial [33,34,35,36,37,38,39,40]. Treatment was well tolerated in only 7% of cases with Grade 3+ acute or late toxicity. The results of this study were hypothesis-generating and paved the way for prospective trials of neoadjuvant SBRT for borderline resectable pancreatic cancer, including the ongoing ALLIANCE (NCT01992705) and Pancreatic Cancer Research Study (NCT01926197).

### 2.2. The Definitive Setting

There have been a number of studies investigating the role of SBRT in the definitive setting [10,12,13,14,16,17,21,22,23,24,25,26]. Most of these were retrospective series and only two were Phase II studies. The first of these two Phase II studies was published by Hoyer et al. in 2005 [16]. It included 22 patients with T1-3N0 LAPC deemed unresectable by a surgeon measuring up to 6 cm in size. The median PTV volume was 136 cm^3^. Patients were treated with SBRT to a total dose of 45 Gy in three fractions. There was no mention of chemotherapy in the trial or a requirement that patients also undergo systemic chemotherapy. Study results were disappointing with only a 57% local control rate at one year and a median survival of 5.4 months. The toxicity profile was unfavorable with 36% of patients experiencing an increase in pain and analgesic use, 23% of patients with severe mucositis or ulceration, and one gastric perforation requiring emergent surgery. There are a number of reasons why this early experience with SBRT yielded such poor outcomes. These include a lack of fiducials for daily set-up and target localization possibly resulting in tumor miss, a lack of OAR constraints, resulting in high toxicity rates, and no specific requirements for systemic chemotherapy in conjunction with SBRT either before or after treatment, which we know today is a key component of treatment for patients with both resectable and especially unresectable disease.

Ten years after the publication of the Hoyer study, Herman et al. published their Phase II trial of SBRT for unresectable pancreatic cancer [21]. In this study, 49 patients with LAPC were treated with three weeks of gemcitabine, followed by a one-week break, followed by SBRT to the tumor to a total dose of 33 Gy in five fractions. The study included 49 patients with a median PTV volume of 71.4 cm^3^. Unlike its predecessor study, the results of this trial were encouraging. The freedom from local progression at one year was 78% at one year as compared to 57% in the Moyer study. The median progression free survival was 7.8 months and the median overall survival was 13.9 months—a significant improvement from 5.4 months as reported in the Moyer trial. Ten percent of patients were ultimately able to undergo surgery after completion of SBRT. Overall survival for these patients ranged from 13.6 to 40.2 months. The primary endpoint of the rate of late (>3 months after SBRT) gastritis, fistula, enteritis, or ulcers of Grade 2+ was only seen in 11% of patients.

The superior outcomes reported in the Herman et al. trial as compared to the earlier Moyer trial can be attributed to a number of factors. First, unlike in the Herman trial, and all ongoing protocols, fiducial markers were not required to ensure accurate tumor positioning, which may have led to tumor miss. Second, there was no requirement for gemcitabine-based systemic chemotherapy, unlike in the more recent study, which has been shown to improve survival in patients with locally advanced disease [41,42,43]. Furthermore, the Moyer trial did not specify OAR constraints, which likely explains the poor toxicity profile. Lastly, tumor margins were large (5 mm axially and 1 cm cranio-caudal) and there was no dose reduction for overlap with the duodenum and stomach as was done in the Herman trial, which likely explains the 20% rate of severe mucositis and ulceration and the case of gastric perforation requiring surgery.

As radiation techniques have improved and we have learned more about tissue tolerance for high dose radiation therapy, SBRT has emerged as an appealing and highly effective treatment modality for LAPC. A meta-analysis of 19 trials of SBRT for LAPC published in 2017 showed that the median overall survival was greater than 12 months in the vast majority of studies and was particularly favorable in the subset of patients who became resectable after receiving SBRT [20]. These studies have paved the way for randomized Phase III trials (NCT01926197), which will hopefully establish the role of SBRT for LAPC.

### 2.3. The Adjuvant Setting

SBRT in the adjuvant setting for resected pancreatic cancer has not been established. However, there is an ongoing Phase II study examining the role of SBRT in resected T3 and N1 patients, which is currently accruing (NCT02461836).

## 3. SBRT for Hepatocellular Cancer

Hepatocellular carcinoma (HCC) is the second leading cause of cancer death worldwide [44]. The incidence of HCC is approximately equal to the mortality rate, highlighting the aggressive nature of this fatal malignancy. While relatively uncommon in the Unites States (US), it is the fastest growing cause of cancer death in the US [45]. Like with pancreatic cancer, surgery is considered the only curative treatment but most patients either have unresectable disease due to tumor extent or are inoperable due to underlying liver dysfunction. For the latter group of patients, transplant is the ultimate goal because it can cure both the HCC and the underlying liver disease. However, given the limited supply of healthy livers available for transplant, most patients are unable to undergo the procedure right away, thus requiring local treatment as a bridge to liver transplant when an organ becomes available. SBRT for HCC is, therefore, emerging as a safe and effective treatment modality both in the definitive setting for unresectable cancers and as a bridge to transplant for those with underlying liver disease awaiting organ allocation.

Historically, EBRT has been used with caution in the management of hepatic malignancies due to the low tolerance of the liver for radiation [45]. The challenges facing EBRT for HCC have been delivering a sufficiently high dose of radiation to achieve tumor control in an organ that is highly sensitive to radiation and that moves substantially with breathing, making target localization very difficult. Modern-day SBRT techniques make it possible for external beam radiation to overcome these road-blocks by delivering high doses of radiation in a very conformal manner while accounting, and controlling, for tumor motion. Due to a lack of Level I evidence, SBRT is not currently considered a standard treatment for HCC; however, a number of prospective trials have been completed which show its safety and efficacy in the management of this disease [46,47,48,49,50].

The most notable of these prospective trials was a combined analysis of sequential Phase I and II trials conducted at Princess Margaret Hospital published in 2013 [48]. The study included 102 patients with Child–Turcotte–Pugh Class A disease who were unsuitable for other local liver-directed therapy. Median gross tumor volume was 117 mL with a range of 1.3–1913.4 mL. Patients were treated with 30–54 Gy in six fractions, delivered every other day. The treatment was relatively well-tolerated with Grade 3+ toxicity reported in 30% of cases. Local control at one year was 87% and overall survival was 55%.

SBRT has also been combined with trans-arterial chemo-embolization (TACE) with favorable outcomes in the literature so far. There are a number of advantages to combining SBRT with TACE. TACE can shrink tumors, thus creating a smaller treatment volume for SBRT. The combination of the two treatments allows for ablation of vascular components of the tumor with TACE, while the poorly vascularized, necrotic portions can be targeted by SBRT. Finally, SBRT can be used to recanalize tumors with arterial or portal vein thromboses, rendering TACE more effective. A retrospective study of patients with tumors ≥3 cm compared outcomes among patients who received TACE plus SBRT compared to TACE alone [51]. The authors found that, after censoring for liver transplantation, overall survival was significantly better with TACE plus SBRT compared to TACE alone (33 vs. 20 months, respectively).

In conclusion, modern-day SBRT techniques allow for safe and effective delivery of external beam radiation therapy for hepatocellular carcinoma. While more data is needed, available evidence shows that there is a role for radiation in the management of this disease. Specifically, radiation plays a role for lesions unsuitable for other local therapies, for larger lesions in which TACE is less effective, and in cases with portal vein thrombosis in which other therapies are contra-indicated or ineffective [52]. Indeed, there is a randomized Phase III study underway comparing treatment with Sorafenib with or without SBRT in patients with HCC (NCT01730937).

## 4. SBRT and Immunomodulatory Biomarkers in Upper GI Malignancies and Therapeutic Implications

In addition to allowing for higher doses of radiation to be delivered to a more precise target in a shorter treatment time, emerging data shows that radiation, specifically SBRT, has advantageous effects on the immune system, which may have therapeutic implications. The immune-stimulatory effects of radiation have been known for some time. Indeed, Demaria et al. first introduced the concept of radiation as an “in situ vaccine” in 2004 [53]. The authors suggest that radiation turns the radiated tumor into a vaccine by priming the immune system to target cancer cells in other sites in addition to treating the disease locally, a concept known as the abscopal effect. Since then, Formenti and colleagues have been able to site clinical data lending credence to this concept in which radiation delivered locally results in tumor response at the site of radiation as well as decreased tumor burden outside the irradiated field, mediated by the patient’s immune system [54,55,56,57].

While the dose and fractionation needed to optimally prime the immune system is not yet known, SBRT appears to be superior to conventional radiation in terms of its effect on tumor immunogenicity. First, SBRT creates less of an immunosuppressive effect on the host’s immune system as compared to conventionally fractionated radiation, both due to the absence of the concomitant chemotherapy and to the relatively smaller volume of irradiated bone marrow [58,59,60]. In addition, emerging data shows that SBRT has the potential advantage of directly increasing tumor immunogenicity [2,61]. Indeed, numerous pre-clinical models summarized very nicely by Popp et al. have shown that SBRT induces complex changes in the tumor microenvironment [53]. The authors review several preclinical studies showing that SBRT leads to increased recruitment of immune cells, including antigen-presenting-cells and dendritic cells, as well as cytokines and chemokines, which are all involved in the immune response [53]. The authors also summarize existing clinical data demonstrating evidence that SBRT mediates the abscopal effect. However, most of the data they cite involves patients with either melanoma or lung cancer, for which the benefit of immunotherapeutic agents is already widely established [62,63,64].

There is limited clinical data addressing the role of SBRT and its effects on the immune system for gastrointestinal malignancies; however, studies are emerging. Specifically, studies have shown an increase in tumor infiltrating lymphocytes and in soluble PD-L1, involved in T-cell regulatory pathways, following SBRT in pancreatic and hepatic malignancies [61,65]. The immunogenic effect of SBRT on tumors, coupled with the absence of an immunosuppressive effect on the patient, may allow for novel therapeutic approaches to treating upper GI malignancies by combining SBRT with immunotherapy. The combination of these two therapies may be more effective than either treatment alone.

Tumors have varying degrees of immune activity based on their histology. The tumor environment in pancreatic cancer, for instance, is considered to be immunosuppressive with a low degree of infiltration by T cells [66]. As a result, immunotherapy with immune check-point inhibitors has been investigated in this disease, with disappointing results [67,68]. As Foley et al. detail in their paper on immunotherapy for pancreatic cancer, removing immune suppression without providing a means to activate the immune system is likely responsible for these disappointing outcomes [66].

Emerging data show that SBRT has more of an immunogenic effect on the tumor environment when compared to conventionally fractionated radiation therapy delivered concurrently with chemotherapy for pancreatic cancer. In a recent presentation at the American Society for Radiation Oncology (ASTRO) annual meeting, Chen et al. presented their data on tumor infiltrating cells in pancreatic cancer [65]. They showed that there was a statistically significant difference in the ratio of CD8 T-cells to FOXP3 T-regulatory cells (CD8/FOXP3) detected in tumor cells following SBRT as compared to conventionally fractionated therapy. They also showed that a higher CD8/FOXP3 ratio was associated with improved progression free survival. The authors thus show that SBRT may be more effective in terms of local control as compared to conventional fractionation, in addition to showing that SBRT is more immunogenic than conventional radiation. The combination of SBRT and immunotherapy may, therefore, provide novel therapeutic strategies for this disease. Indeed, there are studies underway looking at the efficacy of combining SBRT with immunotherapy in the treatment of pancreatic cancer (NCT 02648282).

Hepatocellular carcinoma, on the other hand, has been shown to be an immune active malignancy with a high infiltration of T cells [66]. Kim et al. recently presented their data on the effects of radiation on soluble PD-L1 in hepatocellular carcinoma and its therapeutic implications [61]. The authors showed that radiation therapy increases the expression of PD-L1 on tumor cells. This increase was noted both after conventionally fractionated radiation and after SBRT; however, the levels after SBRT continued to rise one month following treatment, which was not true following conventionally fractionated radiation. The authors conclude that this data may provide evidence for a novel therapeutic strategy for patients with HCC that combines SBRT with PD-L1 blockade.

## 5. Conclusions and Future Directions

SBRT has emerged as a highly promising treatment modality in the management of upper GI malignancies. It allows for more curative doses of radiation to be delivered to a highly conformal treatment volume, in a short amount of time, allowing for effective and expedited treatment for these highly aggressive malignancies.

In the case of pancreatic cancer, SBRT has emerged as an effective neoadjuvant treatment to render tumors which are unresectable upfront, resectable after treatment, thus allowing an increased number of patients to undergo potentially curative resection. In the case of LAPC, SBRT also has the potential advantage of rendering some of these initially unresectable tumors resectable, while also providing reasonable long-term survival rates for patients undergoing definitive treatment. Studies are ongoing for SBRT in the adjuvant setting. In the case of HCC, SBRT has emerged as an effective treatment for patients in which other local therapies either cannot be performed or are ineffective as, for example, for large tumors or those with portal venous invasion.

Finally, the effects of SBRT on the immune system and tumor micro-environment is an area of active research with heretofore promising results. Emerging data shows that SBRT can increase tumor immunogenicity, thus providing a rationale for novel therapeutic approaches combining SBRT and immunotherapeutic agents with the hope that the combination of the two treatments will result in better outcomes than either treatment alone. More data is needed to confirm these initial findings. Prospective trials studying the combination of SBRT and immunotherapy in the management of upper GI malignancies are needed to confirm the therapeutic implications of these retrospective studies, and some are already underway.
